# Preparation and characterization of activated carbon from agricultural wastes and their ability to remove chlorpyrifos from water

**DOI:** 10.1016/j.toxrep.2023.01.011

**Published:** 2023-01-20

**Authors:** Omaima A. Hussain, Amal S. Hathout, Yasmin E. Abdel-Mobdy, M.M. Rashed, E.A. Abdel Rahim, A.S.M. Fouzy

**Affiliations:** a Food Toxicology and Contaminants Department, National Research Centre, Dokki, Cairo, Egypt; b Entomology and Pesticides Department, Faculty of Agriculture, Cairo University, Giza, Egypt; c Chemical Biochemistry Department, Faculty of Agriculture, Cairo University, Giza, Egypt

**Keywords:** Agricultural waste, Activated carbon, Chlorpyrifos, BET, SEM, EDX, FTIR

## Abstract

Chlorpyrifos is an organophosphate insecticide linked to neurological dysfunctions, endocrine disturbance, cardiovascular illness, genotoxicity, histopathological abnormalities, immunotoxicity, and oxidative stress. Therefore, the aim of this study was to prepare activated carbon from agricultural waste to adsorb and remove chlorpyrifos from aqueous solutions, as well as to study the physicochemical characteristics of the prepared activated carbon.Activated carbon was prepared from agricultural waste (banana peels, orange peels, pomegranate peels and date stones). The activated carbon prepared showed an exterior surface that was irregular and full of cavities with Brunauer-Emmett-Teller(BET) surface areas of 94.26, 111.75, 183.89, and 289.86 m^2^/g for activated carbon prepared from orange peels, date stone, pomegranate peels, and banana peels respectively. The Scanning Electron Microscope (SEM) image revealed that the activated carbon's exterior surface was irregular and full of various shapes and sizes of cavities.The Energy Dispersive X-Ray (EDX) indicated the existence of carbon, oxygen, silicon and potassium in banana peels-derived activated carbon, whereas carbon, oxygen, silicon and potassium, in addition to aluminium, were detected in the pomegranate peels-derived activated carbon. The Fourier-Transform Infrared Spectroscopy (FTIR) analysis of prepared activated carbon revealed several functional groups, including carboxylic acid, carbon dioxide, and aromatic compounds. Results also showed that the activated carbon significantly removed chlorpyrifos from water, recording 97.6%, 90.6%, 71.48%, and 52.00 % for activated carbon prepared from pomegranate peels, banana peels, date stones and orange peels, respectively. The study concluded that agricultural waste-derived activated carbon could be employed as an alternative pesticide adsorbent.

## Highlights

• The SEM image revealed that the exterior surface of activated carbon was irregular and full of cavities.
• The EDX examination revealed the existence of carbon, oxygen, and other elements in the prepared activated carbon.
• The FTIR spectra showed the nature of the functional groups existing on the exterior of the activated carbon.
• The pomegranate peel-activated carbon possessed maximum total pore volume and the highest surface area.
• Pomegranate and banana peels-derived activated carbon adsorbed chlorpyrifos pesticide by 97.39% and 90.61%, respectively,

## Graphical Abstract

**Figure:**
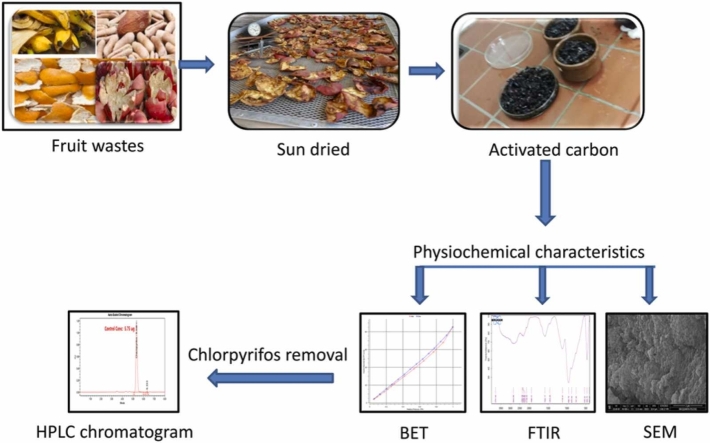

## 1 Introduction

Organophosphorus pesticides play an essential role in pest management, accounting for more than 80% of all insecticides [1]. Chlorpyrifos is an organophosphorus pesticide used in agriculture to control Coleoptera, Diptera, Homoptera, and Lepidoptera in soil and foliage [2]. It is regarded as the active component in many insecticides and is used by farmers and city inhabitants [3], [4], [5]. Chlorpyrifos has been established to remain in the environment [4], [6], [7], and its breakdown products are found in soil and water due to excessive and indiscriminate use, causing considerable toxicity to non-target creatures such as aquatic organisms and humans [8], [9].

Chlorpyrifosis banned as a residential and field pesticide (agricultural insecticide) [10] by the United States Environmental Protection Agency (EPA)due to its adverse effects on human health. Chlorpyrifos is a moderately hazardous chemical that can harm humans' central nervous, circulatory, and respiratory systems [11]. High dosages of chlorpyrifos can cause acute poisoning by covalently blocking acetylcholinesterase (AChE), which overstimulates the nervous system, causing neuromuscular symptoms and, in extreme cases, seizures, respiratory paralysis, and death [12]. Long-term usage of chlorpyrifos can also cause cancer, skin feeling issues, and infertility [13], [14]. Pesticide removal technologies are now available for contaminated water, and they include several methods,like the adsorption process, which has sparked considerable interest due to their ease of use and reusability, as well as their high efficiency and cost-effectiveness [15]. Among the many adsorbents available, activated carbon is essential in reducing environmental pollution due to its vast surface area, porous structure, and capacity to absorb pollutants selectively. It is recognised to have several advantages because of its amphoteric behaviour, which gives materials flexibility [16]. Because commercial activated carbon is expensive, various low-cost options for pollutant removal from the contaminated water have been investigated. These include tea leaves [17], almond shells [18], bean pods [19], rice husks [20], cherry stones [21], olive stones [22], [23], [24], coconut shells [25], and wild sugarcane [26].

The availability of non-renewable and somewhat expensive starting materials like coal, which is an essential financial factor, has limited the utilisation of activated carbon despite its extensive use for wastewater treatment [27]. The novelty of this work is to prepare agricultural waste in the preparation of activated carbon and the ability of this activated carbon to adsorb pesticide residues.Thus, it is necessary to discover new, low-cost materials for preparing activated carbon to study their ability to remove various contaminants. Therefore, the aim of this study was to prepare activated carbon from agricultural waste to adsorb and remove chlorpyrifos from aqueous solutions, as well as to study the physicochemical characteristics of the prepared activated carbon.

## 2 Materials and methods

### 2.1 Chemicals

Dichloromethane, phosphoric acid (85 %), and acetonitrile of HPLC grade were purchased from Merck (Darmstadt, Germany). Chlorpyrifos was purchased from Santa Cruz Biotechnology Inc. (Santa Cruz, CA 95060, USA).

### 2.2 Fruits

We obtained fresh fruits of Washington Navel orange (*Citrus sinensis L*.) and sweet orange from the Egyptian Ministry of Agriculture farms on December 30th, 2020. Dates (*Phoenix dactylifera* L.) were procured from a date farm in El Ayat, Giza,on January 4th, 2021. We purchased pomegranates (*Punicagranatum L*.)and bananas (*Musa* sp.) from the neighbourhood market on October 10th, 2020.

### 2.3 Preparation of fruit peels

Fruit peels were manually separated, cleansed with distilled water to remove the impurities, wiped dry, cut into pieces measuring 2 X 2 cm, and then sun-dried for 96 h at 60 °C.

### 2.4 Preparation of date stones

Date stones were washed with water to remove any attached date flesh and dried in the sun. The gathered date stones were ground in a heavy-duty mill (M20 Universal Mill, IKA®-Werke GmbH & Co. KG, Germany) and filtered through a 0.25 mm mesh.

### 2.5 Preparation of activated carbon

Activated carbon was prepared from various agricultural wastes through a single-step chemical activation process using phosphoric acid (H_3_PO_4_), according to Girgis et al [28]. In brief, crushed fruit peels and date stones (100 g) were soaked in phosphoric acid in a ratio of 1:3 (w/w). The mixture was slightly agitated to ensure penetration of the acid throughout the agricultural wastes, heated to 70°C for 2 h and left overnight at room temperature. After that, the mixture was put in a muffle furnace, and the temperature was raised to 500°C for 2 h at the rate of 5°C/min. while isolated from oxygen. The acid was removed with distilled water until the pH reached 6.8. The activated carbon was then dried at 110°C for 24 h using an electric oven.

### 2.6 Physiochemical characteristics of activated carbon

#### 2.6.1 Scanning Electron Microscope (SEM)

The surface morphology was investigated using the SEM equipped with Energy Dispersive X-ray spectra (EDX) to determine the precise elemental report [29].

### 2.7 Fourier-Transform Infrared Spectroscopy (FTIR) analysis

The FTIR spectra of the prepared activated carbon were recorded between 450 and 4000 cm^−1^using FTIR spectroscopy (Bruker, USA) according to the method described by Hussain et al [30]. and Ettish et al [31]. The prepared activated carbons were dried in an oven at 50–60 °C (at atmospheric pressure) for 3–5 h to guarantee complete water removal.

### 2.8 Brunauer-Emmett-Teller (BET) analysis

According to Tan et al [25]., the adsorption isotherms were conducted from the adsorption of nitrogen at − 196 °Cover the processed activated carbon utilising an automatic adsorption apparatus (Quanta chrome Instruments, Model Nova 3000e series, USA). The BET equation was applied to determine the relative pressure (P/P_O_) within 0.05 – 0.20. The obtained results from the isotherms were used to define the BET surface area. The Horvath–Kawazoe (HK) method was applied to determine the micro-pore volume. The Barrett–Joyner–Halenda (BJH) method defined the pore size division and the mesopore volume.

### 2.9 The ability of activated carbon to remove chlorpyrifos

The adsorption ability of activated carbon against chlorpyrifos was studied. Chlorpyrifos (8 mg) was dissolved in 10 mL acetonitrile and added to a litre of distilled water to reach a concentration of 8 mg/l.Then the prepared chlorpyrifos solution was divided into falcon tubes (50 mL) containing an amount of 0.25 and 0.50 g of agricultural waste activated carbon. The mixture was incubated in a shaker incubator at 100 rpm, 25 °C for 1 h. Then 50 mL of the aqueous solution was extracted twice with 50 mL of dichloromethane using a separating funnel. The mixed extracts were dehydrated by passing through a column of anhydrous sodium sulphate and evaporated using a rotary evaporator to approximately 1 mL. The extracts were then transferred to vials, and the volume was reduced to 0.1 mL using a nitrogen stream for HPLC analysis [5], [31].

### 2.10 Statistical analysis

The results were expressed as mean ± SD. Statistical analysis was performed using the SPSS software version 16. A one-way analysis of variance (ANOVA) was performed, in which *P* < 0.05 was considered statistically significant.

## 3 Results and discussion

### 3.1 Physiochemical characteristics of activated carbon

#### 3.1.1 Scanning electron microscope

**Fig. 1:**
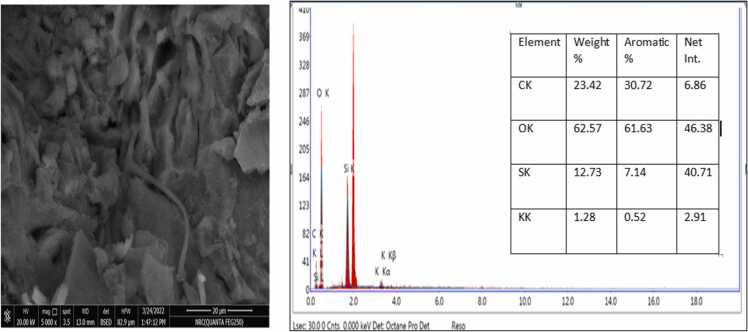
The SEM micrograph and EDX analysis of the banana peels activated carbon.

**Fig. 2:**
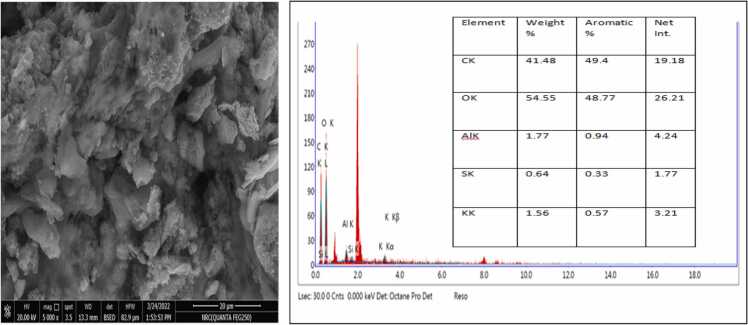
The SEM micrograph and EDX analysis of the pomegranate peels activated carbon.

**Fig. 3:**
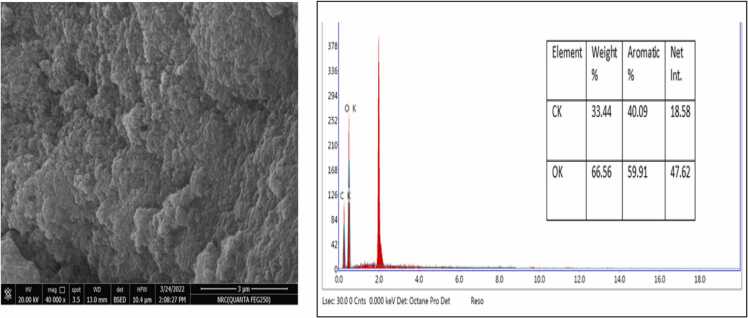
The SEM micrograph and EDX analysis of the orange peels activated carbon.

**Fig. 4:**
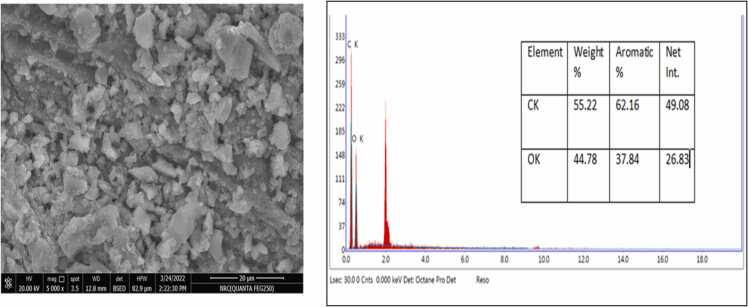
The SEM micrograph and EDX analysis of the date stones activated carbon.

The SEM examined the morphological structure of activated carbon prepared from bananas, pomegranates, orange peels, and date stones (Fig. 1, Fig. 2, Fig. 3, Fig. 4). The SEM image revealed that the activated carbon's exterior surface was irregular and full of various shapes and sizes of cavities. The EDX is an analytical technique used for elemental composition measurement and chemical characterisation of activated carbon.Datain Fig. 1 revealed the existence of carbon, oxygen, silicon and potassium in banana peels derived activated carbon. Similar results were reported for pomegranate peels derived activated carbon, whereas carbon, oxygen, silicon and potassium, in addition to aluminium (Fig. 2. Results in Fig. 3, Fig. 4 only indicated the presence of carbon and oxygen. Similar observations were reported by Blachnio et al [32]., who noted that the chemical composition of the activated carbon is as follows; carbon, oxygen, nitrogen, phosphorus, and silicon, whereas for the other samples, only carbon and oxygen. In our study, neither nitrogen nor phosphorus was detected. Results are also in agreement with Mopoung et al [33]. The silicon content in the banana and pomegranate-activated carbon roughly corresponds to its content in the original fruit peels.

### 3.2 Fourier-transform infrared spectroscopy

**Fig. 5:**
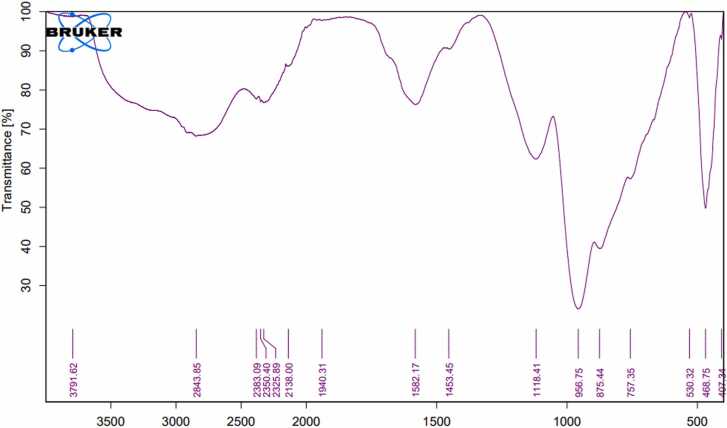
The FTIR analysis of banana peels activated carbon.

**Fig. 6:**
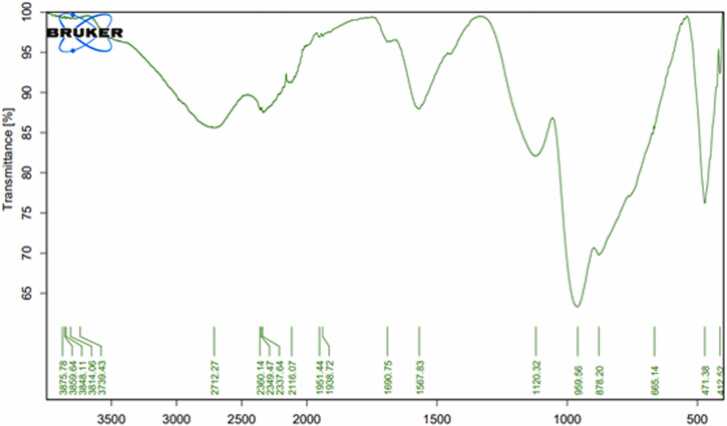
The FTIR analysis of pomegranate peels activated carbon.

**Fig. 7:**
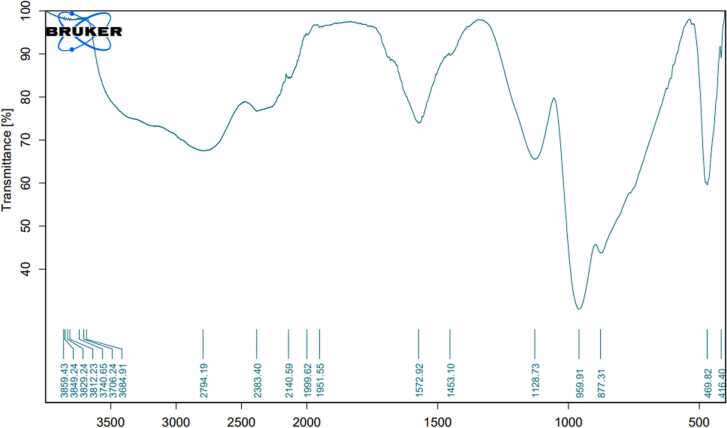
The FTIR analysis of orange peels activated carbon.

**Fig. 8:**
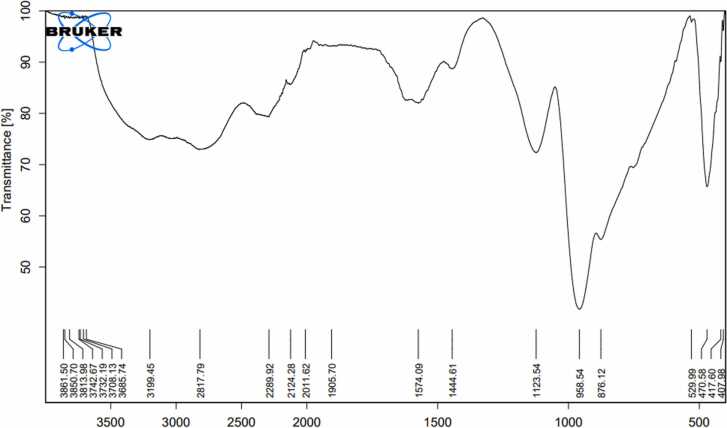
The FITR analysis of date stones activated carbon.

The FTIR spectra of activated carbon prepared from banana, pomegranate, orange peels, and date stones were determined to understand the nature of the functional groups existing on the exterior of the activated carbon (Table 1, Fig. 5, Fig. 6, Fig. 7, Fig. 8). Data in Fig. 5 displayed the peaks that showed the complicated nature of the banana peel-derived activated carbon. Bands appeared at 2843.85, 1940.31, 1582.17, 1453.45, 1118.41, 757.36, and 530.02 cm^−1^ were assigned to O-H stretching, carboxylic acids, C=C=C, allene, C=C stretching, cyclic alkane, C-H bending, alkanes, C-O stretching, aliphatic ether, C-H bending,mono-substituted, and C-Cl stretching, halo compound respectively. On the other hand, the bands 2350.40 & 2325.00 were assigned to O=C=O carbon dioxide.

**Table 1:** FTIR of activated carbon produced from agricultural waste.

IR frequencies (cm^−1^)				Functional groups
Banana peels	Pomegranate peels	Orange peels	Date stones	
-	-	3681.19	3665.45	Alcohol (O-H stretching)
2843.85	2712.2	2794.19	2817.79	carboxylic acid (O-H stretching)
	-	-	-	Carboxylic acids (–OH)
2350.40, 2325.00	2360.4, 2349.47, 2337.64	2383.40	-	Carbon dioxide (O=C=O)
-	-	2140.59	2124.28	carbodiimide (N = C=N stretching)
1940.31	-	1999.62,1951.55	1905.70	Allene (C=C=C stretching)
-	1690.75	-	-	Primary amide (C=O stretching)
1582.17	-	1572.92	-	Cyclic Alkane (C=C stretching)
1453.45	-	1453.10	1444.61	Alkane (C-H bending)
1118.41	1120.32	1128.73	1123.54	Aliphatic ether (C-O stretching)
757.36	-	-	-	Mono-substituted (C-H bending)
-	665.14	-	-	Aklene (C=C bending)
530.02	-	-	529.99	Halo compound (C-Cl stretching)

Functional group identification was based on data obtained from FTIR Functional Group Table with Search-InstaNANO[48]

Meanwhile, Fig. 6 shows the peaks that indicate the complex nature of the pomegranate peel-derived activated carbon. Bands appeared at 2712.2, 1690.75, 1120.32, and 665.14 and were assigned to O-H stretching, carboxylic acid, C=O stretching, primary amide, C-O stretching, aliphatic ether, and C=C bending, alkene respectively. Meanwhile, the bands 2360.4, 2349.47, & 2337.64 were assigned to O=C=O, carbon dioxide.

On the other hand, Fig. 7 data revealed the functional groups of the orange peel-derived activated carbon. Results indicated that the bands appeared at 3681.19, 2794.19, 2383.40, 2140.59, 1572.92, 1453.10, and 1128.73, which were assigned to O-H stretching, alcohol, O-H stretching, carboxylic acid, O=C=O, carbon dioxide, N = C=N stretching, carbodiimide, C=C stretching, cyclic alkane, and C-H bending, alkane, respectively, whereas both 1999.62, &1951.55 were assigned to C=C=C stretching, allene.

Results in Fig. 8 showed the functional groups present on the date stone-derived activated carbon, whereas bands appeared at 3665.45, 2817.79, 2124.28, 1905.70, 1444.61, 1123.54, and 529.99 were assigned to O-H stretching, alcohol, O-H stretching, carboxylic acid, N = C=N stretching, allene, C-H bending, alkane, C-O stretching, aliphatic ether, and C-Cl stretching, halo compounds.

Results concerning the banana peels derived activated carbon were similar to those reported by Zheng and Wang [34], whereas some peaks were identical, while others were quite different. Similar observations were reported by Viena et al [35]. FTIR data for pomegranate peel-derived activated carbon were in agreement with Ali et al [36]., who indicated that pomegranate activated carbon has three clear peaks at 3400, 1600, and 1205. Similarly, some FTIR peaks were similar to Al-Onazi et al [37]., whereas others differed. For orange peel-derived activated carbon, results are similar to those of Gul et al [38]., who showed identical peaks. Results differed from Deshmukh et al [39]., who found four major absorption bands at 1900–2100 cm^−1^, 1200–1300 cm^−1^, and 750–850 cm^−1^, ^1^ for orange peels activated carbon. Also, for date stones derived from activated carbon, results differed from Erhayem et al [40]., who detected the following functional groups: C=O, -COOH, C-N and C=C. Results were also different from Sahmarani et al [41]., who reported that bands appeared at 1562 cm^−1^, from 900 to 1300 cm^−1^, and 1070–1080 cm^−1^, while two bands appeared at 743 and 875 cm^−1^.

### 3.3 Brunauer-Emmett-Teller

**Fig. 9:**
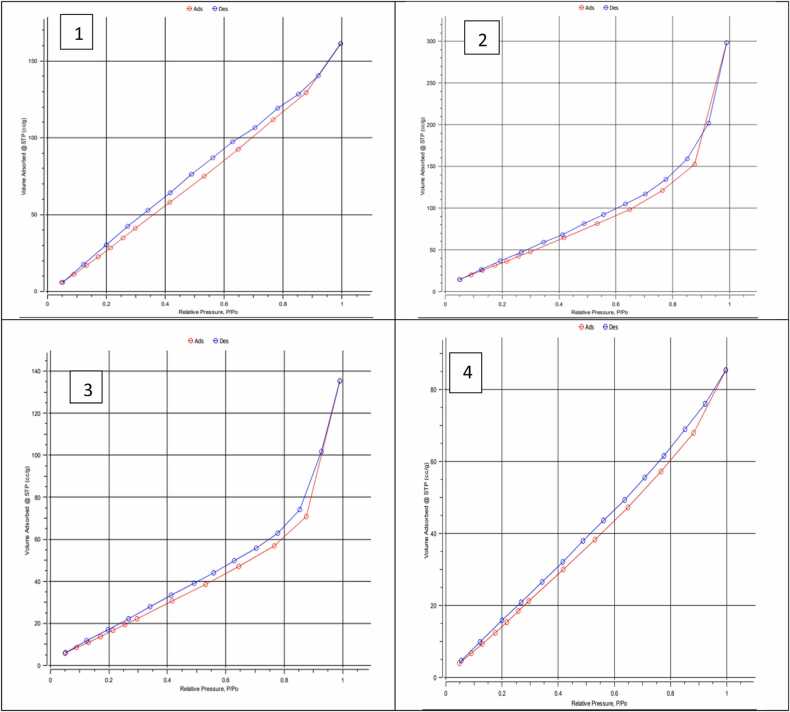
Nitrogen adsorption-desorption isotherms of activated carbon prepared from 1)banana peels, 2)pomegranate peels,3) orange peels, and 4) date stones. The upper lines are the desorption of each component.

The N_2_ adsorption-desorption isotherms were used to measure the prepared activated carbon's specific surface area and porous structure. Different techniques, such as BET and BJH, are used to calculate the surface area of the prepared activated carbon (Table 2). The surface area (BET) and porosity were the main indications of the perfect preparation conditions.The textural properties of the activated carbonare shown in Fig. 9, which indicates that by increasing relative pressure, the volume adsorbed increases.The surface area (S_BET_) and total pore volume (V_T_) of the prepared activated carbonare affected by the type of agricultural waste used. Although the activated carbon based on banana peel showed a maximum (S_BET_of 289.86 m^2^/g), the pomegranate peel activated carbon possessed maximum total pore volume (V_T_) (0.462 cm^3^/g) with the highest BJH surface area (125.11 m^2^/g). The orange peel-derived activated carbon showed a lower BET surface area (94.26 m^2^/g), while date stone-derived activated carbon possessed a lower total pore volume (0.132 cm^3^/g). According to the ratio S_BJH_/S_BET_ and V_BJH_/V_BET_, it is clear that the mesoporosity character increased with pomegranate and orange peels derived activated carbon. The average pore radius of these prepared activated carbon results were 5.07 and 4.45 nm for pomegranate and orange peels derived activated carbon.

**Table 2:** The textural properties, surface area and porevolume, and pore radiusof the prepared activated carbon.

Activated carbon	Surface Area (m^2^/g) (S_BET_)	Surface Area (m²/g) (S_BJH_)	% (S_BJH_/S_BET_)	Pore volume (cm^3^/g) (V_BJH_)	Total pore volume (cm3/g) V_T (0.95)_	Pore radius Dv (r) (nm)	% (V_BJH_/V_T_)	Average Pore radius (n)
**Banana peels**	289.86	113.348	39.1016	0.218175	0.249	1.92882	87.5502	1.7239e
**Pomegranate peels**	183.89	125.113	68.0352	0.428014	0.462	1.93886	92.6407	5.0258e
**Orange peels**	94.264	58.7282	62.3016	0.192906	0.209	1.93051	92.3445	4.4484e
**Date stones**	111.75	57.8684	51.7834	0.115743	0.132	1.93326	87.8788	2.3669e

Similarly, Ettish et al [31]. noted that the surface area of the synthesised activated carbon from cinnamon was high, with a high pore volume.These results agree with Mella et al [24], who demonstrated that the raw material gave extended carbon content, high volatile material, and low-ash content, which served as a great candidate for the development of activated carbon.Many studies have focused on the characteristics of activated carbon prepared from various organic solid wastes, whereas in this study, the BET surface area of activated carbon prepared from date stone was higher than other activated carbon prepared from olive stones (83.72 m^2^/g) [42]. Similar results were reported by Abdul Khalil et al [43]., who noted that the S_BET_ external surface area was 199.16, 140.99 and 71.49 (m^2^/g) for oil palm empty fruit bunch, bamboo stem and coconut shell, respectively. For banana peel-derived activated carbon, the S_BET_ results were considered higher than those of Zheng and Wang [35].

### 3.4 The ability of activated carbon to remove chlorpyrifos

Data in Table 3 showed the adsorption capacity of chlorpyrifos pesticide by different fruit waste-derived activated carbon. Results revealed that 0.25 g pomegranate and banana peels-derived activated carbon removed chlorpyrifos by 97.39% and 90.61%, respectively. On the other hand, 0.25 g orange peels and date stones-derived activated carbon removed chlorpyrifos by71.48% and 52.00%, respectively. It was observed that orange peels and date stones-derived activated carbon removed lower amounts than pomegranate and banana peels-derived activated carbon. This might be due to the high surface area of pomegranate and banana peels-derived activated carbon which could have led to high adsorption capacity,

**Table 3:** Percentage of chlorpyrifos removal from water.

Activated carbon	Quantity of activated carbon added (g)	Chlorpyrifos concentration (µg/mL)	Percentage of removal (%)
Control	-	5.75^a^**±** 0.12	-
Banana peels	0.25	0.54^g^**±** 0.03	90.61
	0.50	1.12^f^**±** 0.07	80.52
Pomegranate peels	0.25	0.15^h^**±** 0.02	97.39
	0.50	2.52^c^**±** 0.10	56.17
Orange peels	0.25	1.64^e^±0.08	71.48
	0.50	1.96^d^**±** 0.09	65.91
Date stones	0.25	2.76^b^**±** 0.09	52.00
	0.50	1.03^f^**±** 0.08	82.09

Results are mean ± SE

Within each raw number, superscripts with different letters are significantly different,*P* ≤ 0.05 (LSD= 0.24)

Results showed that fruit waste-derived activated carbons at lower concentrations removed a higher amount of chlorpyrifos, except for date stones-derived activated carbon,whereas higher activated carbon concentrations removed higher amounts of chlorpyrifos concentrations. That might be due to the saturation that could have happened ata concentration of 0.25 g.

Although there are various methods for removing pesticides from water, adsorption on activated carbons, on the other hand, is the most efficient and extensively used method for removing pesticides from water streams [44]. This might be due to their large surface area, high adsorption capacity, and some surface functional group content-often providing carbon surface characteristics that enhance adsorption.The dispersion force between the π electrons in the pesticide structure and the π electrons on the surface of the carbon material is predicted to be the significant factor in pesticide adsorption on carbon materials [45].

According to many factors, including the source of the activated carbon, surface characterisation, particle size, and functional groups, the adsorption capabilities of various adsorbents differed. Similar findings were reported by Ettish et al [31]., who claimed that cinnamon stick-derived activated carbon was a promising adsorbent for removing chlorpyrifos. On the other hand, activated carbon made from oily sludge successfully removed phenol from an aqueous solution [46]. Similarly, Alam et al [47]. reported that acid red 4 and methylene blue were removed from aqueous media by activated carbon prepared from the wood of *Paulownia tomentosa*.

## 4 Conclusion

Activated carbon from fruit waste (date stones, bananas, pomegranate, and orange peels) might be a promising adsorbent for removing chlorpyrifos from water. This study could be considered a new contribution to the production of activated carbon from fruit -waste. The outcomes of this research could be a guide for the practical use of fruit waste to remove pesticides from contaminated water. Carbon from fruit waste may be utilized in adsorption columns to produce safe water. Further investigations on the disposal of used carbon are required to ensure that it does not result in further environmental issues like the release of pesticides into the air, water, and soil.

## CRediT authorship contribution statement

**Omaima A. Hussain:** Conceptualization, Methodology. **Amal S. Hathout:** Writing – review & editing. **Yasmin E. Abdel-Mobdy:** Visualization, Data curation. **M.M. Rashed:** Supervision. **E.A. Abdel Rahim:** Supervision. **A.S.M. Fouzy:** Project administration, Funding acquisition.

## Declaration of Competing Interest

The authors declare that they have no known competing financial interests or personal relationships that could have appeared to influence the work reported in this paper.

## Data Availability

Data will be made available on request.
